# Biochemical and Molecular Dynamics Study of a Novel GH 43 α-l-Arabinofuranosidase/β-Xylosidase From *Caldicellulosiruptor saccharolyticus* DSM8903

**DOI:** 10.3389/fbioe.2022.810542

**Published:** 2022-02-11

**Authors:** Md. Abu Saleh, Shafi Mahmud, Sarah Albogami, Ahmed M El-Shehawi, Gobindo Kumar Paul, Shirmin Islam, Amit Kumar Dutta, Md. Salah Uddin, Shahriar Zaman

**Affiliations:** ^1^ Microbiology Laboratory, Department of Genetic Engineering and Biotechnology, University of Rajshahi, Rajshahi, Bangladesh; ^2^ Department of Biotechnology, College of Science, Taif University, Taif, Saudi Arabia; ^3^ Department of Microbiology, University of Rajshahi, Rajshahi, Bangladesh

**Keywords:** β-xylosidase, α-l-arabinofuranosidase, *Caldicellulosiruptor saccharolyticus*, biochemical characterization, molecular dynamics

## Abstract

The complete hydrolysis of xylan can be facilitated by the coordinated action of xylanase and other de-branching enzymes. Here, a GH43 α-l-arabinofuranosidase/β-xylosidase (CAX43) from *Caldicellulosiruptor saccharolyticus* was cloned, sequenced, and biochemically investigated. The interaction of the enzyme with various substrates was also studied. With a half-life of 120 h at 70°C, the produced protein performed maximum activity at pH 6.0 and 70°C. The enzyme demonstrated a higher activity (271.062 ± 4.83 U/mg) against *para* nitrophenol (*p*NP) α-L-arabinofuranosides. With xylanase (XynA), the enzyme had a higher degree of synergy (2.30) in a molar ratio of 10:10 (nM). The interaction of the enzyme with three substrates, *p*NP α-L-arabinofuranosides, *p*NP β-D-xylopyranosides, and sugar beet arabinan, was investigated using protein modeling, molecular docking, and molecular dynamics (MD) simulation. During the simulation time, the root mean square deviation (RMSD) of the enzyme was below 2.5 Å, demonstrating structural stability. Six, five, and seven binding-interacting residues were confirmed against *p*NP α-L-arabinofuranosides, *p*NP β-D-xylopyranosides, and arabinan, respectively, in molecular docking experiments. This biochemical and *in silico* study gives a new window for understanding the GH43 family’s structural stability and substrate recognition, potentially leading to biological insights and rational enzyme engineering for a new generation of enzymes that perform better and have greater biorefinery utilization.

## Introduction

Xylan, the most prominent hemicellulose in plant biomass, is made up of a repeating β1, four xylose residue backbone with modified galacturonic acid or acetyl group, rhamnose, and other substituents that vary depending on the other sources (Yen et al., 2015). Although the metabolic process and the molecular mechanism underlying the biosynthesis of xylan are not fully understood, β-d-xylosidase works as a rate-limiting enzyme to hydrolyze xylan (Wierzbicki et al., 2019; Xu et al., 2019). Xylose is coupled to the arabinose substituents *via* α-1,2- or α-1,3-glycosidic linkages (De Vries and Visser, 2001).

Arabinose, the second most abundant pentose in nature, is found in varieties of plant cell wall polymers. Arabinoxylan, arabinan, and arabinogalactan are arabinose-containing polysaccharides that are significant components of plants’ cell walls. The first chain of this heteropolysaccharide is created from β-1,4-linked d-xylopyranosyl sugar with l-arabinose scattered randomly. Furthermore, the backbone of arabinan and arabinogalactan consists of a linear 1,5-linked l-arabinofuranosyl polymer and 1,6-glycosidically connected d-galactopyranose residues, respectively (Liu et al., 2021).

Arabinoxylan degradation requires the coordinated action of endo-1,4-β-xylanases (EC 3.2.1.8), α-l-arabinofuranosidase (EC 3.2.1.55), α-glucuronidase (EC 3.2.1.139), acetyl (xylan) esterase (EC 3.1.1.72), ferulic acid esterase (EC 3.1.1.73), and β-xylosidase (EC 3.2.1.37) (De Vries et al., 2000; Sørensen et al., 2003). Side-chain substitution of l-arabinofuranoside is additionally found in xylans, and also, the alteration of those side chains can inhibit enzymatic degradation by xylanases and pectinases (Shallom et al., 2002; Rahman et al., 2003). The α-l-arabinofuranosidase catalyzes the hydrolysis of a terminal non-reducing α-1,2-, α-1,3-, and α-1,5-l-arabinofuranosyl residues from arabinoxylan and other polysaccharides containing l-arabinose (Saha 2000; Sozzi et al., 2002; Zhang et al., 2021). This enzyme acts in tandem with endo-1,5-α-L-arabinanases to convert polysaccharides or l-arabinose oligosaccharides to l-arabinose. l-arabinose holds back sucrase and glucose increase caused by sucrose consumption (Osaki et al., 2001; İlgü et al., 2018). As a result, it has the power to scale back obesity and forestall disorders linked to high glucose.

CAZy classified arabinofuranosidases in seven distinct glycoside hydrolase families (GH 1, 3, 10, 43, 51, 54, and 62), for knowing their structural and mechanical activities and evolutionary relationship according to the similarities of their protein sequences (Limsakul et al., 2021). Compared with most active catalytically potent xylosidases, GH 43 is categorized into GH-F clan for having no transglycosylation activity (Jordan and Li, 2007). According to the CAZy database (http://www.cazy.org), three putative β-xylosidases were assigned to this GH family. Various arabinofuranosidases from both fungal and bacterial origins have recently been found, offering essential information for understanding the enzymes. Thermotolerant biomass-degrading enzymes often perform well due to their higher stability and potentiality, and thermophilic bacteria are one of the finest sources of arabinofuranosidases (Hu et al., 2018; Long et al., 2020).

Although only a few publications deal with β-xylosidase from a couple of bacterial strains, no paper was found for β-xylosidase from *Caldicellulosiruptor saccharolyticus* DSM8903 (Xu et al., 2019). *C. saccharolyticus* is a thermophilic bacterium; hence, the enzyme produced by it would be thermostable. This enzyme can act in extreme environmental conditions and is crucial for biotechnological operations that require harsh settings (Friedricht and Antranikian, 1996). Furthermore, higher temperatures result in faster reaction speeds, reduced contamination risk, and enhanced substrate solubility (Béguin and Aubert, 1994). Thus, thermotolerant α-l-arabinofuranosidase/β-xylosidase has a great deal of commercial potential (Zurawski et al., 2015). GH families have varied substrate specificities for arabinofuranosidases. Individual members’ substrate specificity could vary; the GH 51 and 54 AFs had a wide range of substrate specificity (Yang et al., 2015).

It is difficult to determine the regularity in the substrate specificity of enzymes with cost and labor intensity since a sufficient amount of xylosidase enzymes are required to conduct multiple tests. Furthermore, for mass production of the enzyme, biological stability, and characterization are critical (Ebert and Pelletier, 2017). Bioinformatics is becoming a prominent technique in this regard, since it provides user-friendly algorithms for gaining insights into protein–substrate interactions in a short amount of time and at a low cost (Ebert and Pelletier, 2017). Autodock is a software program that can be used to determine substrate specificity. In this study, we investigated the structural stability, substrate specificity, and potential of the GH43 family enzyme using biochemical and *in silico* methods, leading to biological insights and rational enzyme engineering to develop a new generation of enzymes.

## Materials and Methods

### Bacterial Strains, Plasmid, and Culture Conditions

*C. saccharolyticus* DSM8903 was used as a source of genomic DNA. The expression vector *pEASY* blunt E_2_ was used to produce a recombinant enzyme with six His-tags in its carboxy-terminus. Trans1-T1 phage-resistant chemically component cells and *E. coli* BL21 (C43) were employed for recombinant plasmid maintenance and protein production, respectively. The recombinants were cultured in Luria-Bertani (LB) medium supplemented with ampicillin (100 μg/ml) at 37°C. *C. saccharolyticus* DSM8903 genome was obtained from Molecular Microbial Engineering Group, Qingdao Institute of Bioenergy and Bioprocess Technology, China. Vector was obtained from TransGen Biotech Co. Ltd., China. All the used substrates were purchased from Megazyme; plasmid extraction kit and PCR purification kit from QIAGEN Shanghai Co. Ltd; and SDS, APS, DNA marker, protein marker, DNA loading dye, TEMED, etc. were purchased from Promega United States. All other used chemicals were of analytical grade.

### Cloning and Expression

The gene (1586; *C. sac*-0359)-encoding region was amplified by PCR using pfu DNA polymerase for expression and purification of the α-l-arabinofuranosidase/β-xylosidase from *C. saccharolyticus* in *E. coli.* The primers were designed so that they could be cloned in-frame into the *pEASY* blunt E_2_ expression vector.

Recombinant vector (*pEASY* blunt E_2_ and CAX43) (Supplementary Figure S1) was transformed to Trans1-T1 component cells and poured on LB agar with ampicillin (100 μg/ml). Ampicillin-resistant clones were chosen, and therefore, the accuracy of the inserts was confirmed by sequencing. Plasmid was extracted by using a TransGen plasmid purification kit and transformed into BL21 (C43) for protein expression.

### Induction and Purification of Protein

After overnight incubation, one colony was injected in 10 ml of LB liquid and shaken at 37°C. The pre-culture was then transformed to a 1-L LB medium with ampicillin (100 μg/ml) and incubated at 37°C. When the optical density at 600 nm reached 0.6 to 0.8, isopropyl-beta–d-thiogalactopyranoside (IPTG) was added at a concentration of 0.3 mM to induce protein, and also, the cells were shaken for an additional 7 h at 30°C.

The cells were collected after centrifuging (4,000×*g*) at 4°C for 30 min and resuspended to 15 ml washing buffer (Tris—30 mM and NaCl—500 mM, pH—8). By ultrasonication, the cells were then disrupted, and debris was removed by centrifugation (7,500 rpm) for 30 min at 4°C. Since the proteins expressed by *Caldicellulosiruptor* sp. are thermostable, the recombinant proteins were heated in an exceeding water bath at 70°C for 15 min before being chilled on ice. The cells were again centrifuged at 4°C (7,500 rpm for 15 min) to precipitate the other co-eluting thermostable host proteins. After loading the supernatant onto a nickel-nitrilotriacetic acid (Ni-NTA)-sefinose column (Sangon, Shanghai, China), it was washed with washing buffer and binding buffer (Tris—30 mM, NaCl—500 mM, and imidazole—40 mM; pH—8), then eluted with elution buffer (Tris—30 mM, NaCl—500 mM, and imidazole—300 mM; pH-8). By using a 10-kDa cutoff membrane, the active fractions were identified and reconstituted with protein storage buffer (Tris—30 mM, NaCl—100 mM, and 15% glycerol; pH—7). SDS-PAGE was carried out to separate the purified protein, and the Bradford method was used to determine the concentration with bovine serum albumin as a standard (Bradford, 1976).

### Enzyme Activity Assay

The activity of α-l-arabinofuranosidase and β-xylosidase was measured using *p*NP α-l-arabinofuranosides and *p*NP β-D-xylopyranosides as substrates, according to the Beer–Lambert law. At optimum pH and temperature, one unit of enzyme activity was defined as the quantity of enzyme that released 1 µmol of *p*-nitrophenol per minute. The reactions were as follows: 100 µl of an enzyme (0.0002 mg/ml) in citric acid phosphate buffer (pH 6) and 100 µl of 2 mM substrate in citric acid phosphate buffer (pH 6) were mixed and heated at 70°C for 10 min; 1 ml of 0.2 mM Na_2_CO_3_ was added to stop the reaction, and the absorbance was measured at 415 nm (Margolles and de los Reyes-Gavilin, 2003).

### The Effect of pH and Temperature on the Enzyme Activity

The optimum pH for α-l-arabinofuranosidase/β-xylosidase activity was measured by incubating at 70°C for 10 min in citric acid phosphate buffer with pH ranging from 4 to 8. The effect of temperature on enzyme activity was also measured in the same buffer (pH 6) at different temperatures ranging from 40°C to 90°C. The findings were reported as relative activity (%).

By incubating the enzyme solutions at various temperatures (60°C, 70°C, 75°C, and 80°C), the half-life of thermal inactivation of the enzyme was determined as a function of incubation time. At various periods, aliquots of samples were extracted at appropriate intervals, and the activity of enzyme was assessed immediately. The time it took for the relative activity on the best-fitting line to decline by half was the enzyme’s half-life.

### Substrate Specificity and Kinetic Parameters

The substrate specificity of purified enzyme was also tested using *p*NP α-L-arabinofuranosides, *p*NP β-D-xylopyranosides, and a variety of different polysaccharides (1%) like beechwood xylan, insoluble wheat arabinoxylan, sugar beet arabinan, and debranched sugar beet arabinan. In the case of *p*NP α-L-arabinofuranosides and *p*NP β-d-xylopyranoside, the activity was measured by above description, but when using 1% polysaccharides (beechwood xylan, insoluble wheat arabinoxylan, sugar beet arabinan, and debranched sugar beet arabinan), the reaction mixtures (6 μg enzyme and substrate) were incubated at 70°C for 14 h, and therefore, the reducing sugar was measured by the dinitrosalicylic acid method (Miller, 1959) with xylose and l-arabinose as a regular. Kinetic parameters of purified enzyme were examined against *p*NP α-L-arabinofuranosides with various concentrations (from 0.5 to 3.5 mM/l). By employing non-linear regression, *K*_m_ (mmol/L) and *k*_cat_ (/sec) were measured from the Michaelis–Menten equation with GraphPad Prism nine software (San Diego, CA) (Su et al., 2012).

### Determination of Synergistic Effects

The enzyme XynA (xylanase, isolated from the *C. saccharolyticus*) was employed to explore the synergistic effects of the enzyme. The reactions were allotted at pH 6 and 70°C in citrate buffer. After incubation, the mixture was centrifuged, and therefore, the reducing sugar within the supernatant was quantified using the DNS technique (Miller, 1959). The synergy calculated supported the ratio between the reducing sugars liberated by the combined actions of enzymes to the sum of the sugar liberated by each enzyme (Iakiviak et al., 2011; Yang et al., 2016).

### Ligand Preparation

The 3D structure of the *p*NP α-L-arabinofuranosides, *p*NP β-D-xylopyranosides, and arabinan was collected from the PubChem database (Kim et al., 2019). The structure was energy minimized using Avogadro software package in mmff94 force field with steepest gradient algorithms (Halgren, 1996).

### Protein Preparation

The enzyme’s 3D structure has yet to be explored, and we used a homology modeling approach to anticipate the hypothetical model structure of the protein. The sequence was obtained from NCBI (Federhen, 2012) with the accession number (ABP66000.1) and entered into Phyre2 tools (Kelley et al., 2015) as Fasta format. The D chain of bifunctional GH43-CE from *Bacteriodes eggerthii* (PDB ID: 6MLY) was used as a template. The alignment coverage was 96% (13–522), and the confidence score was 100. The protein model was energy minimized in a short molecular dynamics simulation. After the minimization process, the accuracy and geometry of the model protein were checked in Ramachandran plot (Sheik et al., 2002), ERRAT (Hasan et al., 2015), and Verify3D (Eisenberg et al., 1997) model. The model structure was further used for docking and molecular dynamics simulation.

### Active Site Prediction

The active sites of the modeled protein were predicted from the Computed Atlas of Surface Topology of protein (CASTp) webserver (http://sts.bioe.uic.edu/castp), which provides the pockets on the protein surface by molecular surface and solvent-accessible surface models. The webserver can predict the active sites/catalytic sites precisely along with the surface and functional features of the protein (Binkowski et al., 2003).

### Molecular Docking

The docking study was conducted to search out the interaction dynamics of the glycosidase hydrolase and ligand molecules. The docking study was employed in AutoDock Vina (Trott and Olson, 2010). The ligand was converted into the PDBQT format because AutoDock Vina (Trott and Olson, 2019) only accepts this format. The grid box’s center was *X*: 67.996, *Y*: 11.53, and *Z*: 21.38 Å, whereas the scales were *X*: 230, *Y*: 139, and *Z*: 161 Å. The binding energy of the docked complex was calculated in kilocalorie/mole unit. Also, the ligand molecules and protein were separated from PDB ID: 6MLY by the Discovery Studio and docked in AutoDock Vina to compare with the other docked complexes. The docked pose was combined in Pymol package (DeLano, 2002), and the interactions were also investigated in Discovery studio (Miyata, 2015).

### Molecular Dynamics

The docked complexes were simulated by YASARA dynamics to analyze the conformational variety and stability of the complexes in AMBER14 force field. The systems were initially cleaned and hydrogen bonds were optimized. The linear constraint solver (LINCS) was used to limit all bond lengths, and SETTLE was employed to constrain water molecules. SHAKE algorithms were used to fix the chemical bond length in hydrogen bonds. The initial energy-minimization approach was carried out using the simulated annealing method by steepest gradient algorithms (5,000 cycles) (Krieger and Vriend, 2015). In all cases, a cubic simulation cell was formed with periodic boundary conditions by extending 20 Å and TIP3P water model with a solvent density of 0.997 g/cm^3^. The acid equilibrium constant for each amino acid within the protein was computed during the solvation process. The SCWRL algorithms were used in conjunction with hydrogen bonding network optimization to keep each amino acid residue in the correct protonation state. The Berendsen thermostat kept the simulation temperature constant. The system was neutralized by adding 0.9% NaCl, pH 7.4, at 36.85°C temperature. The long-range electrostatic interaction was calculated using particle meshes Ewald algorithms with a cut-off radius of 8 Å (Krieger et al., 2006). The time was set as 2.0 fs for simulation. Finally, the simulation was run for 100 ns using the Berendsen thermostat and constant pressure, with each trajectory saved after a 100-ps interval. Root mean square deviation, root mean square fluctuation, a radius of gyration, solvent accessible surface area, hydrogen bond, and MM-PBSA were all calculated using simulated trajectories (Swargiary et al., 2020; Mahmud et al., 2021a; Mahmud et al., 2021b; Mahmud et al., 2021c; Mahmud et al., 2021d; Mahmud et al., 2021e; Mahmud et al., 2021f).

### Statistical Analysis

For each biological sample, three independent replications were used in all studies. In SPSS statistics 20 software, the significance of each group’s data was analyzed statistically at *p* ≤ 0.05 using one-way ANOVA followed by Duncan’s multiple range test (DMRT). Confidence of interval (CI) of the value was calculated using the following formula: CI = mean ± SE. *Z* / √*n*. Here, SE is for sample standard error; *n* stands for sample size; *Z* stands for confidence level value. Graphical figures were prepared using GraphPad Prism 8.

## Results

### Gene Cloning and Sequencing

PCR was used to extract a 1,586-kb gene fragment from *C. saccharolyticus*. This gene encodes 528 amino acids, predicting a molecular weight of 60.59 kDa. We investigated the phylogenetic relationship (Supplementary Figure S2) of this enzyme with other GH43 family enzymes from different bacterial species. The deduced amino acid had 99.05% similarity with the GH43 from *Caldicellulosiruptor* sp. F32*.* No signal peptide was predicted by using the signal IP-5. Based on substrate specificity, the α-L-arabinofuranosidases have been classified into two glycoside hydrolase families (EC 3.2.1.55 and EC 3.2.1.99) and to five glycoside hydrolase families, 3, 43, 51, 54, and 62 based on sequence homology.

### Expression, Purification, and Biochemical Characterization of Recombinant Protein

**FIGURE 1:**
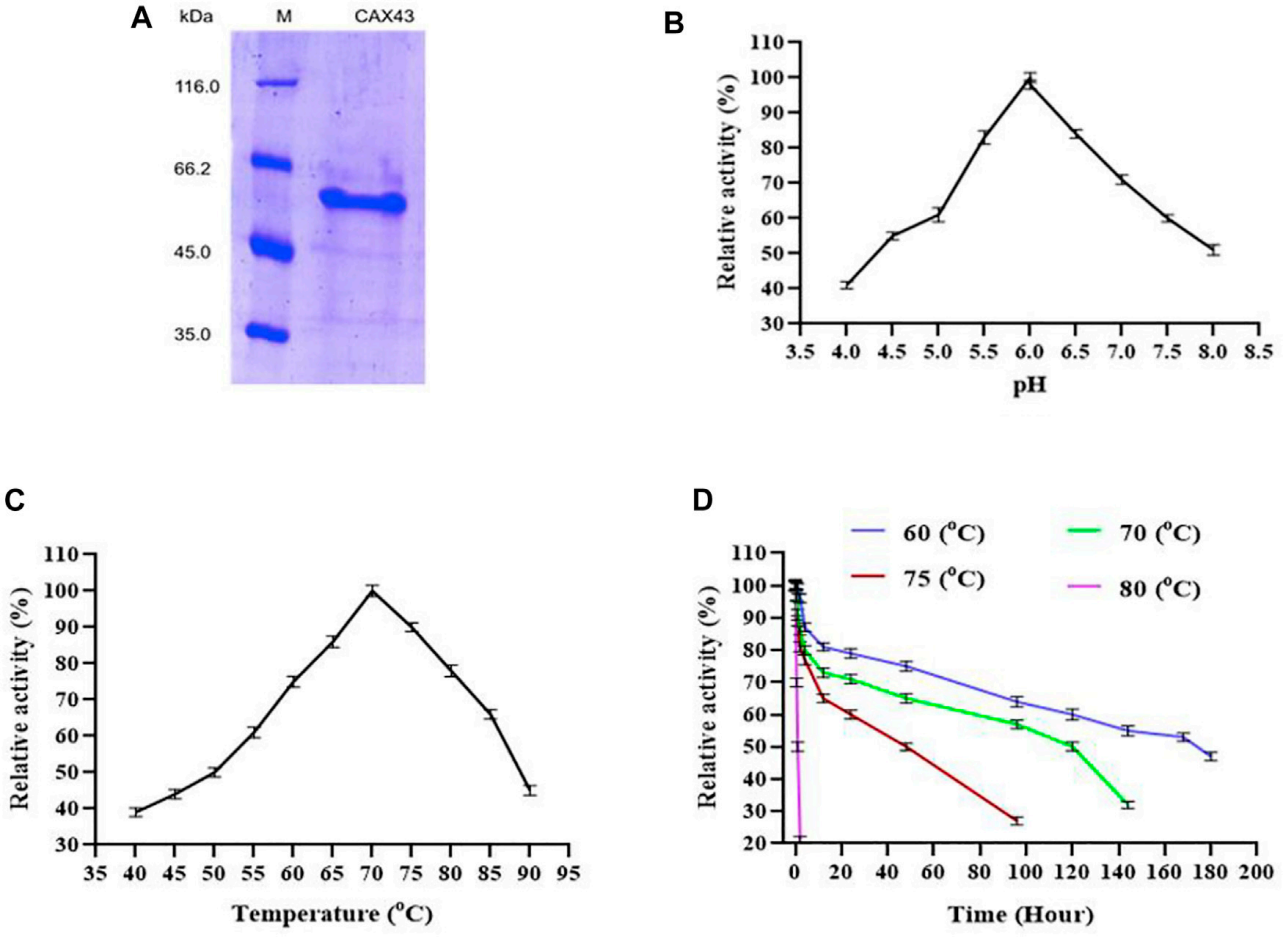
Production and characterization of CAX43. **(A)** SDS-PAGE analysis of the purified recombinant protein. Lanes: M, the standard protein molecular weight markers; 1, the recombinant protein purified by Ni^2+^-NTA affinity chromatography. **(B)** The effect of pH, **(C)** the effect of temperature, and **(D)** thermal inactivation at different temperatures. All the reactions were conducted in citric acid phosphate buffer and *p*NP α-L-arabinofuranosides as a substrate.

For purification of recombinant protein, a histidine tag was appended to the c-terminus of the protein. This His-tagged protein was purified using a nickel affinity chromatography (Figure 1A) and demonstrated a molecular weight of 60.59 kDa. The biochemical characteristics of purified α-l-arabinofuranosidase/β-xylosidase were investigated using *p*NP α-L-arabinofuranosides and β-D-xylopyranosides as a substrate at 70°C. The enzyme activity was examined by covering the range between pH 4 to eight at 70°C. CAX43 showed its highest level of activity at pH 6 (Figure 1B). The activity of the recombinant enzyme was also examined at different temperatures ranging from 40°C to 90°C, and the higher activity was recorded at 70°C (Figure 1C). To observe the thermostability, the enzyme was incubated at 60°C, 70°C, 75°C, and 80°C for various periods with *p*NP α-L-arabinofuranosides as a substrate. The enzyme had a half-life over 160 h at 60°C, but at 80°C, the enzyme lost its activity rapidly (Figure 1D).

The enzyme was active against different synthetic (pNP α-L-arabinofuranosides and β-D-xylopyranosides) and natural (wheat arabinoxylan, sugar beet arabinan, and debranched sugar beet arabinan) substrates. The specific activity of the enzyme (U/mg) was 271.061 ± 4.83, 111.822 ± 3.23, 0.693 ± 0.08, 2.672 ± 0.83, and 1.061 ± 0.33 for pNP α-L-arabinofuranosides, pNP-β-d-xylopyranoside, wheat arabinoxylan, sugar beet arabinan, and debranched sugar beet arabinan, respectively. The kinetic parameters of the recombinant enzyme were determined by using pNP α-L-arabinofuranosides. The Michaelis–Menten constant (*K*_m_), turn over number (*k*_cat_), and the catalytic efficiencies (*k*_cat_/*K*_m_) of the enzyme were 1.071 mmol l^−1^, 234.674 s^−1^, and 219.323 m (mol l^−1^)^−1^s^−1^, respectively.

### Substrate Specificity and Interaction With an Endo-Xylanase

CAX43 exhibited a wide range of substrate specificity. The enzyme’s ability to liberate sugar from beechwood xylan, wheat arabinoxylan, sugar beet arabinan, and debranched arabinan was tested for up to 14 h, and the results are presented in Supplementary Figure S3. The highest activity was observed against sugar beet arabinan. The enzyme had a lower activity against beechwood xylan and wheat arabinoxylan than sugar beet arabinan and debranched arabinan.

**FIGURE 2:**
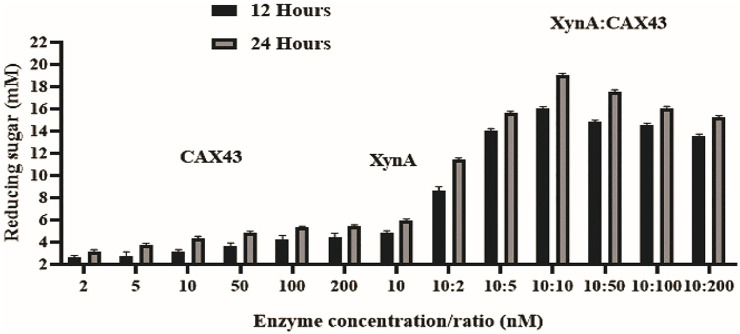
Synergistic effects of CAX43 with XynA. A series of enzyme combinations (molar ratio) were used. All reactions were performed at pH 6 and 70°C against wheat arabinoxylan for two different time durations (12 and 24 h). Reducing sugars were measured by DNS method.

The dinitrosalicylic acid method was used to investigate the synergistic effects of CAX43 with an endo-xylanase (XynA) against wheat arabinoxylan (WAX) (Miller, 1959). Various molar ratios were used to investigate the synergistic effects (Figure 2). The higher (corresponding to a score of 2.301) degree of synergy (DOS) was observed with XynA and CAX43 in a ratio of 10:10 (Table 1). The confidence of interval of these values was 1.604 and 2.221.

**TABLE 1:** Synergistic effect of XynA and CAX43 in various combinations (nM) on wheat arabinoxylan. Data were recorded after 24 h of incubation.

Enzyme ratio (nM) XynA/CAX43	Amount of sugars released (mM) ± SD	Degree of synergy
10:2	11.403 ± 0.295	1.228^a^
10:5	15.310 ± 0.335	1.820^b^
10:10	18.806 ± 0.310	2.301^d^
10:50	17.606 ± 0.203	2.212^d^
10:100	15.311 ± 0.401	2.036^c^
10:200	14.703 ± 0.500	1.866^b^

*Different letters indicate significant differences between mean ± SD of treatments (*n* = 3) at a *p* < 0.05 significance level.

### Protein Modeling and Active Site Prediction

**FIGURE 3:**
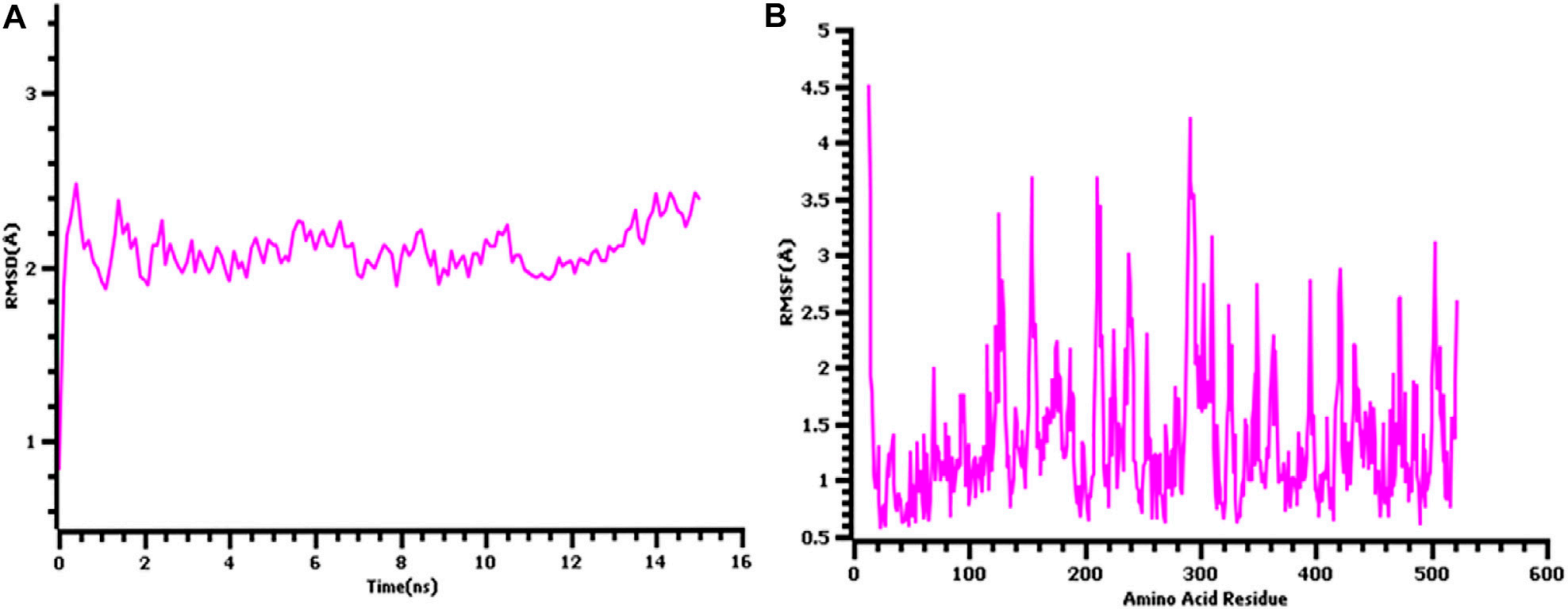
The quality assessment of the hypothetical protein **(A)** root mean square deviation of the model protein and **(B)** the root mean square fluctuation of the hypothetical model.

**FIGURE 4:**
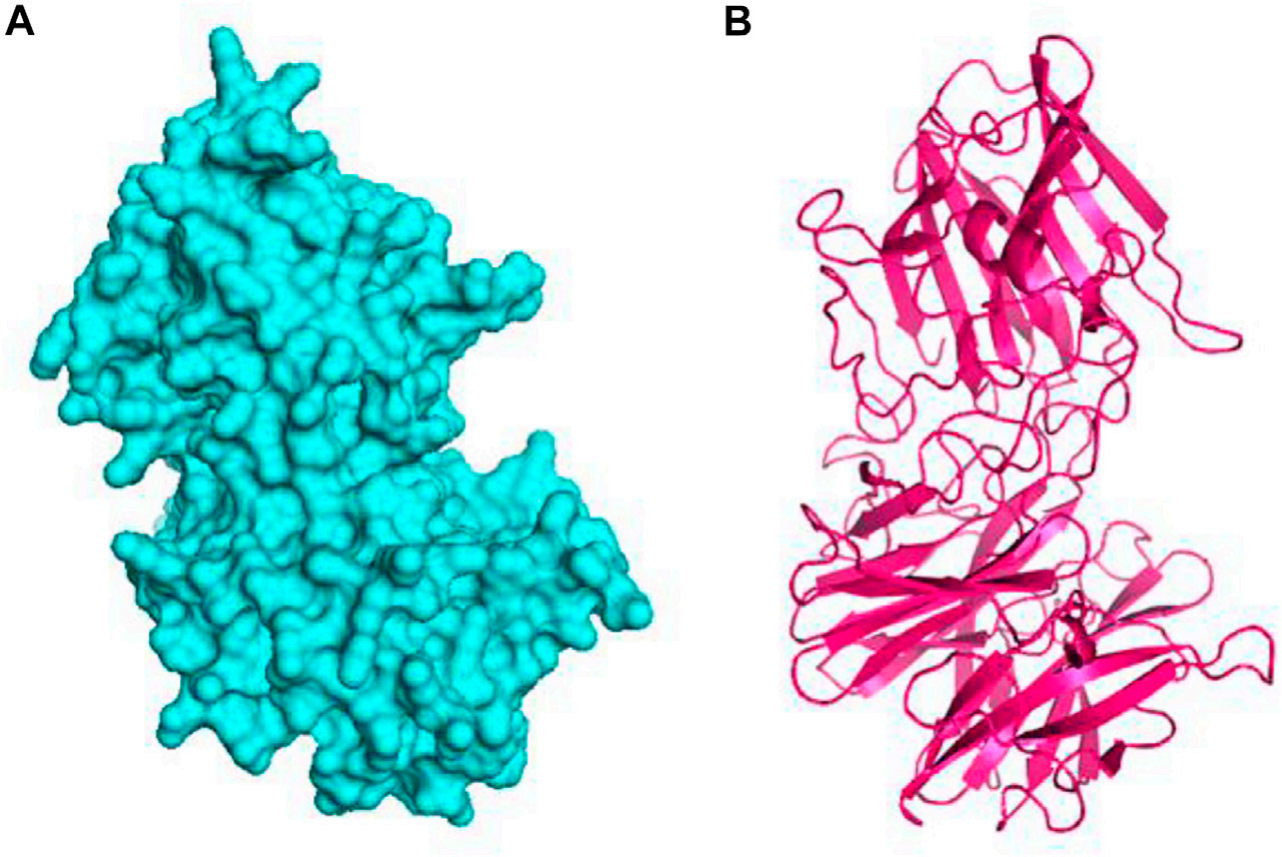
The hypothetical model protein of glycoside hydrolase **(A)** surface view of the predicted protein and **(B)** cartoon view of the protein model.

The 3D structure of the hypothetical model of the enzyme was predicted from Phyre2 tools. The protein model was energy minimized in brief dynamics simulation, where the secondary structure (helix, sheet, turn, and coil) from the hypothetical protein maintained its integrity (Supplementary Figure S4A). The root mean square deviation of the C-alpha atoms had a steady profile and did not deviate excessively. The root mean square deviation (RMSD) was below 2.5 Å during the whole simulation period, which indicates the conformational stability of the hypothetical protein (Figure 3A). The root mean square fluctuation of the amino acid residues of the model protein had a lower RMSF profile for maximum amino acid, which denotes the proteins’ stable nature (Figure 3B). Therefore, the ERRAT score of the minimized protein was greater than 80%, indicating the structural accurateness of the model protein (Supplementary Figure S4B). Moreover, Ramachandran plot analysis of the protein model revealed that 97% of residues were in the core region, which coincides with the complex accuracy (Supplementary Figure S5). We chose this structure for further investigation based on the projected protein model’s quality rating (Figure 4A, B).

The CASTp webserver predicts the active sites of the modeled protein, and about 56 catalytic sites were obtained from the server. Those active points are Phe43, Asn44, Cys45, Val46, Gly48, Val67, Glu68, Arg69, Leu70, Pro71, Ser72, Pro73, Glu74, Tyr75, Thr77, Pro78, Gln79, Ile80, Lys82, Gly83, Ile84, Trp85, Ala86, Ser101, Met102, Pro103, Asp104, Gly130, Ile132, Asp133, Pro134, Ala149, Phe150, Ala151, Phe157, Ser159, Thr189, Glu191, Trp334, His335, Ala336, Asn337, Pro338, Gln339, Asn364, Ile369, Phe370, Phe371, Met372, Pro373, Asn374, Leu375, Ile405, Phe496, Cys497, and Ile498.

### Molecular Docking

**FIGURE 5:**
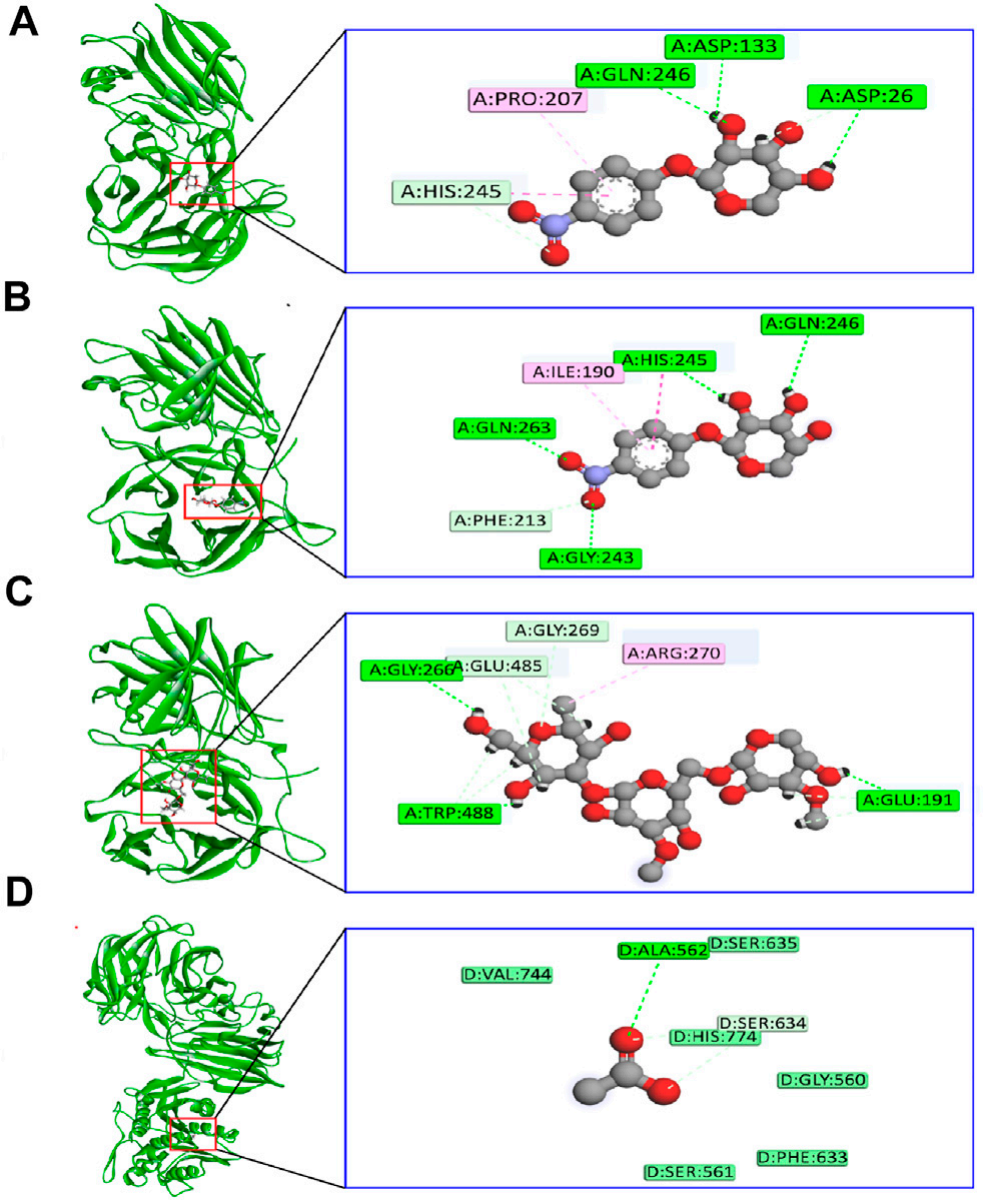
The binding interactions of the ligand molecules; control system; and protein **(A)** *p*NP α-L-arabinofuranosides, **(B)** *p*NP β-D-xylopyranosides, **(C)** arabinan, and **(D)** control system and protein complex.

The binding interactions of protein and ligand complexes were studied. The *p*NP α-L-arabinofuranosides, *p*NP β-D-xylopyranosides, and arabinan had a binding energy of −8.7 kcal/mol, −7.8 kcal/mol, and −8.3 kcal/mol, respectively (Table 2). The control system had −7.6 kcal/mol energy in the molecular docking study. The *p*NP α-L-arabinofuranosides had three hydrogen bonds at the Gln246, Asp133, and Asp26 positions; one carbon hydrogen bond at His245; and one pi-alkyl bond at the Pro207 position (Figure 5A). This compound interacts in the active site of the modeled protein at Asp133 position. *p*NP β-D-xylopyranosides formed four hydrogen bonds at the Gly243, Gln263, His245, and Gln246 positions; one carbon hydrogen bond at the Phe213 position, and one pi-alkyl bond at the Ile190 position (Figure 5B). The arabinan had three hydrogen bonds at the Glu191, Trp488, and Gly266 positions; two carbon hydrogen bonds at the Gly269 and Glu485 positions; and one pi-alkyl bond at the Arg270 position (Figure 5C), whereas it interacts in the active site of the modeled protein at the Glu191 position. Moreover, the control system had one hydrogen bond at the Ala562 position and one carbon hydrogen bond at the Ser634 position (Figure 5D).

**TABLE 2:** The binding interactions of the docked complexes. The binding residues were analyzed in Discovery Studio software package.

Complex	Residues	Bond type	Distance (Å)	Energy (Kcal/mol)
*p*NP α-L-arabinofuranosides	Asp26	Hydrogen bond	2.46	−8.7
	Asp133	Hydrogen bond	2.70	
	Glu191	Hydrogen bond	2.76	
	Gly209	Hydrogen bond	3.09	
	Ile190	Pi-alkyl	5.21	
	Pro207	Pi-alkyl	4.67	
*p*NP β-D-xylopyranosides	Gln246	Hydrogen bond	2.16	−7.8
	Glu191	Hydrogen bond	2.34	
	Gly214	Hydrogen bond	2.95	
	Asp133	Hydrogen bond	2.36	
	Gly209	Hydrogen bond	2.94	
	His245	Pi–Pi stacked	4.89	
Arabinan	Ile487	Hydrogen bond	2.92	−8.3
	Glu485	Hydrogen bond	2.31	
	Asp133	Hydrogen bond	2.77	
	Gly266	Hydrogen bond	3.06	
	Gly81	Hydrogen bond	2.85	
	Gly269	Hydrogen bond	2.49	
	Arg270	Hydrogen bond	3.08	

### Molecular Dynamics

The dynamics simulation was conducted to confirm the docking and structural stability of the complexes (Supplementary Figure S6). The root mean square deviations of the complexes were depicted in Supplementary Figure S6A. The RMSD value of the three docked complexes had an initial upper trend from 0 to 30 ns, but they did not rise sharply. The RMSD profile of these complexes tends to be stable at that phase and maintain the constancy. *p*NP α-L-arabinofuranosides and *p*NP β-D-xylopyranosides had more RMSD profile at that phase than arabinan, but they did not fluctuate much. This trend correlates with the structural stability of the complexes.

Therefore, the solvent-accessible expanse of the complexes was analyzed (Supplementary Figure S6B) to know the change within the protein surface area, where a higher SASA profile indicated the expansion in protein volume and a lower SASA profile indicates the truncated nature of the protein. Although *p*NP-L-arabinofuranosides had a reduced surface area throughout the remainder of the simulation time, the SASA values of the three complexes followed a similar pattern from 0 to 20-ns simulation times. Although the complex had a fluctuation in SASA profile, it was small, and the complex’s profile remained steady. The *p*NP β-D-xylopyranosides had a higher SASA profile than other complexes, indicating that the complex had expanded in volume. The arabinan experienced some changes as well, but they did not diverge significantly in SASA.

The radius of gyration of the complexes was analyzed (Supplementary Figure S6C) to grasp the complex’s liability and motions. The arabinan had a lower RG profile than the other two complexes during the entire simulation time. This trend is related to the structural compactness of the other two complexes. The *p*NP α-L-arabinofuranosides and *p*NP β-D-xylopyranosides had a higher RG profile than arabinan as they are more mobile in simulating conditions. However, these two complexes had lower deviation, indicating that they were more rigid. Moreover, the hydrogen bond of the system defines the stable nature of the complex, and any change in hydrogen bond number can lead to flexibility (Supplementary Figure S6D). The three complexes had less hydrogen bond at the start, but they had a gentle trend within the entire simulation trajectory.

The root mean square fluctuations of the complexes indicate the flexibility across the amino acid residues of the complexes. From Supplementary Figure S7, the RMSF profile of the complexes was much lower than most of the amino acid residues. The maximum amino acid residues exhibited an RMSF value of less than 2.5 Å, except Glu13 (2.70 Å), Arg154 (4.05 Å), Ile155 (2.92 Å), Glu176 (2.91 Å), Lys212 (3.21 Å), Thr186 (2.54 Å), Thr239 (2.53 Å), Asp291 (2.72 Å), Gly292 (3.38 Å), Lys310 (2.61Å), Lys367 (2.64), Lys503 (3.226Å), and Phe522 (2.62Å), indicating less flexibility.

## Discussion

All *Caldicellulosiruptor* species are extremely thermophilic and purely anaerobic, allowing them to degrade plant biomass without any physical and chemical intervention (Yang et al., 2009). Knowing the functions of novel cellulolytic enzymes is crucial for reaching higher quantities of fermentable sugars (Blumer-Schuette et al., 2008). The GH43 is a large family with over 10,000 sequences organized into 37 subfamilies. The CAZy database contains three enzymes from *C. saccharolyticus* that have the GH43 domain. Bifunctional activity with both β-xylosidase and α-l-arabinofuranosidase activity has also been reported (Mai et al., 2000; Canakei et al., 2007; Morana et al., 2008).

Over 80°C, α-L-arabinofuranosidases from *Thermotoga maritima* (Miyazaki, 2005), *Sulfolobus solfataricus* (Morana et al., 2007), and *Clostridium thermocellum* (Taylor et al., 2006) exhibited higher activity. The half-lives of α-L-arabinofuranosidases from *T. maritima* (Miyazaki, 2005), *S. solfataricus* (Morana et al., 2007), *Thermotoga thermarum* DSM 5069 (Xie et al., 2016), and *Thermobacillus xylanilyticus* (Debeche et al., 2002) were 2,·7, 2, and 2 h, respectively at 90°C. However*, C. saccharolyticus* depicted a half-life of 49 h at 75°C (Lim et al., 2010). A multimodular XynF containing GH43 from *C. saccharolyticus* was described (Saleh et al., 2017) and demonstrated maximal activity at pH 6.5 and 70°C temperature. In this study, the recombinant enzyme exhibited higher activity at pH 6.0 and 70°C temperatures. Among all the tested substrates, the enzyme exhibited higher activity against the synthetic substrate *p*NPA. The enzyme demonstrated no activity against beechwood xylan and had a reduced level of activity against wheat arabinoxylan. The result reflects that CAX43 is more effective against synthetic substances than natural ones, which is justified by previous research (de Camargo et al., 2018). Sugar beet arabinan and debranched arabinan contain alpha-1–2, 1–3, and 1–5 glycosidic bonds. CAX43 showed 2.52 times higher activity against sugar beet arabinan than debranched arabinan. This result indicated that it preferred α 1–2 and α 1–3 bonds over α 1–5 bonds.

Prior researches showed that combining hydrolytic enzymes was a beneficial strategy for enhancing sugar releases at a low cost (Taylor et al., 2006; Canakei et al., 2007; Morana et al., 2007; Limsakul et al., 2021). Synergic action was often assessed because the combined effect was greater than the sum of the individual effects. CAX43 was incubated with one extracellular protein XynA from *Caldicellulosiruptor* sp. in various combinations of molar ratio since the molar ratio is important for successful saccharification (Kim et al., 2014). CAX43 showed a substantial synergistic impact against wheat arabinoxylan when incubated with XynA. Among different molar ratios, 10:10 exhibited a higher DOS. This DOS is significantly different from other DOS values except 10:50 ratio (Table 1). Similar results were obtained when XynF was combined with XynA against wheat arabinoxylan (Saleh et al., 2017). Moreover, Mroueh et al. (2019) combined RcAbf62Am2,3 and RcAbf43Ad2,3 with arabinoxylan and got a synergistic factor of 1.53. However, endoxylanases are unable to cleave substrates containing large amounts of arabinoxylan without prior or simultaneous incubation with arabinofuranosidases (Sornyotha et al., 2007; Teeravivattanakit et al., 2017; Mroueh et al., 2019). So, the synergistic potentiality of the CAX43 may open a new dimension in this regard.

Different indicators were used to assess synergy in various systems. In protein–protein interaction, synergy is defined by the value of. DOS > 1 indicates synergism, DOS < 1 indicates antagonism, and DOS = 1 indicates indifference, but in the case of plant extracts and antibiotics interaction, synergy is suggested by FIC index (Silva et al., 2019). FIC between 0.5 and 1 indicates synergy, FIC ≥ 4.0 indicates antagonism, and between 4.0 and 1.0 indicates indifference.

As the substrates include over 30 different 4-nitrophenol (*P*NP)-glycosides, oligosaccharides, and polysaccharides, traditional substrate-specificity analysis for arabinofuranosidase is time-consuming and expensive. Nowadays, molecular mechanisms of substrate binding and catalysis are deciphered using structure-based molecular dynamics simulations, allowing for the rational design of the enzyme to improve its catalytic efficiency and stability (Chen et al., 2015; Yang et al., 2015). Here, we performed an all-atom molecular dynamics simulation for confirming the docking stability of the docked complexes. Also, the docked complexes interacted at the active sites of the protein, which is crucial for the targeted inhibitions or disrupting the function of the target protein. Therefore, the binding energy of the control was lower than the other three complexes, which indicates much better favorable binding of the ligand molecules. Our findings revealed that all three docked complexes are stable with upper trends of RMSD value within 0–30 ns. Molecular dynamics (MD) simulations of β-xylosidase from *Thermomyces lanuginosus* also showed stable nature in 20 ns (Gramany et al., 2016). During the simulations, the radius of gyration (Rg) study revealed the protein’s compactness (Lobanov et al., 2008). Even though arabinan had a lower Rg profile than the other two substrates, all of the complexes were stiff. At pH 7, β-xylosidase showed substantial fluctuations between residues 150 and 200 (Gramany et al., 2016), but CAX43 enzyme showed reduced flexibility across the board. The surface area of the complexes was also analyzed where the stable and lesser degree of fluctuations were observed for the complexes indicating the lower degree of changes in the surface area. Therefore, the hydrogen bond pattern was also similar in the simulation trajectories, which defines the rigid conformations of the docked complexes. The flexibility across the amino acid residues was also observed for very small number of residues, which defines the stable nature, and maximum residues had lower RMSF than 2.5 Å.

## Conclusion

In this study, a GH43 α-l-arabinofuranosidase/β-xylosidase from thermophilic *C. saccharolyticus* DSM8903 was cloned and purified. Our findings show that it can adapt to harsh environments while also possessing desired features such as increased specific and synergistic activity. Molecular dynamics simulation studies revealed major information on binding this enzyme with respective substrates. This study also indicated similar and higher stability in the form of protein–ligand complexes. The docked complexes were validated through the stable profile from the multiple simulation descriptors of the simulation trajectories. As a result, it can be used in the industrial production of l-arabinose in conjunction with endoarabinanase and/or xylanase. The combined findings suggest this enzyme is stable and that it could be used for rational enzyme engineering for improved biorefineries.

## Data Availability

The original contributions presented in the study are included in the article/Supplementary Material; further inquiries can be directed to the corresponding author.
