# Supplementary material for: Biochemical and Molecular Dynamics Study of a Novel GH 43 α-l-Arabinofuranosidase/β-Xylosidase From Caldicellulosiruptor saccharolyticus DSM8903
Source: Front Bioeng Biotechnol. 2022 Feb 11;10:810542. doi: 10.3389/fbioe.2022.810542 (PMC8881100; doi:10.3389/fbioe.2022.810542)

Biochemical and molecular dynamics studyof a novel GH 43 α-L-arabinofuranosidase/ β-xylosidase from *Caldicellulosiruptor saccharolyticus* DSM8903

Md. Abu Saleh[1*], Shafi Mahmud[1], Afaf A Aldahish[2], Gobindo kumar Paul[1], Shirmin Islam[1], Amit Kumar Dutta[3], Md. Salah Uddin[1] and Shahriar Zaman[1]

[1]Microbiology Laboratory, Department of Genetic Engineering and Biotechnology, University of Rajshahi, Rajshahi-6205, Bangladesh.

[2]Department of Pharmacology and Toxicology, College of Pharmacy, King Khalid University, Abha 62529, Asir, Saudi Arabia

[3]Department of Microbiology, University of Rajshahi, Rajshahi-6205, Bangladesh.

*Corresponding author: E-mail: saleh@ru.ac.bd;

Cell: +8801716731747

Fax No. +880721750064

Fig. S1: Model structure of recombinant plasmid using pEASY blunt E_2 vector. Structure was drawn using Snapgene viewer.

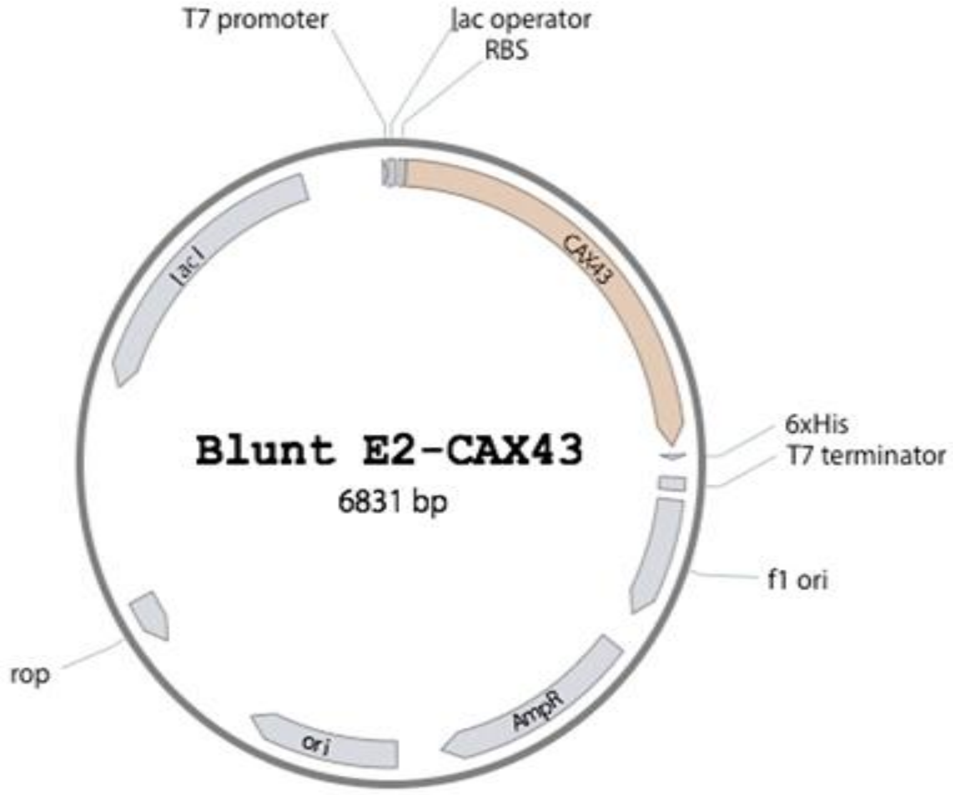

Fig. S2: Phylogenetic tree representing a hypothesis of evolutionary relationships for GH43 in different species of bacteria. Trees were constructed using MEGA 5.05 by the Neighbor-Joining method with 1000 bootstrap replicates, and accession number of each protein sequence were given after of each species name.

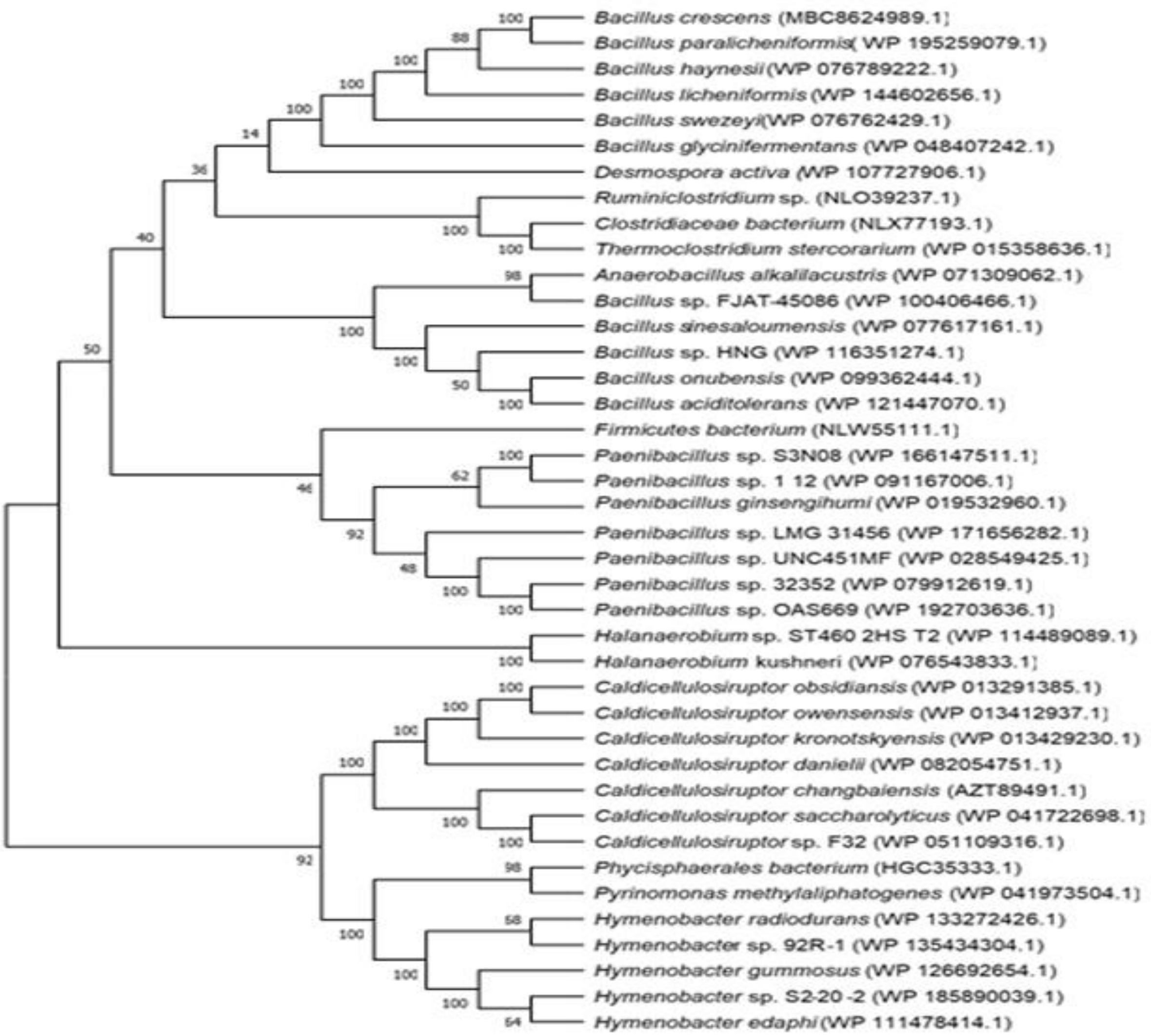

Fig. S3. Hydrolysis product of Beechwood Xylan, Wheat Arabinoxylan, Sugarbeet Arabinan and Debranched arabinan by α–L–arabinofuranosidase/ β-xylosidase. Reactions were performed at 70 ˚C for 14 hours.

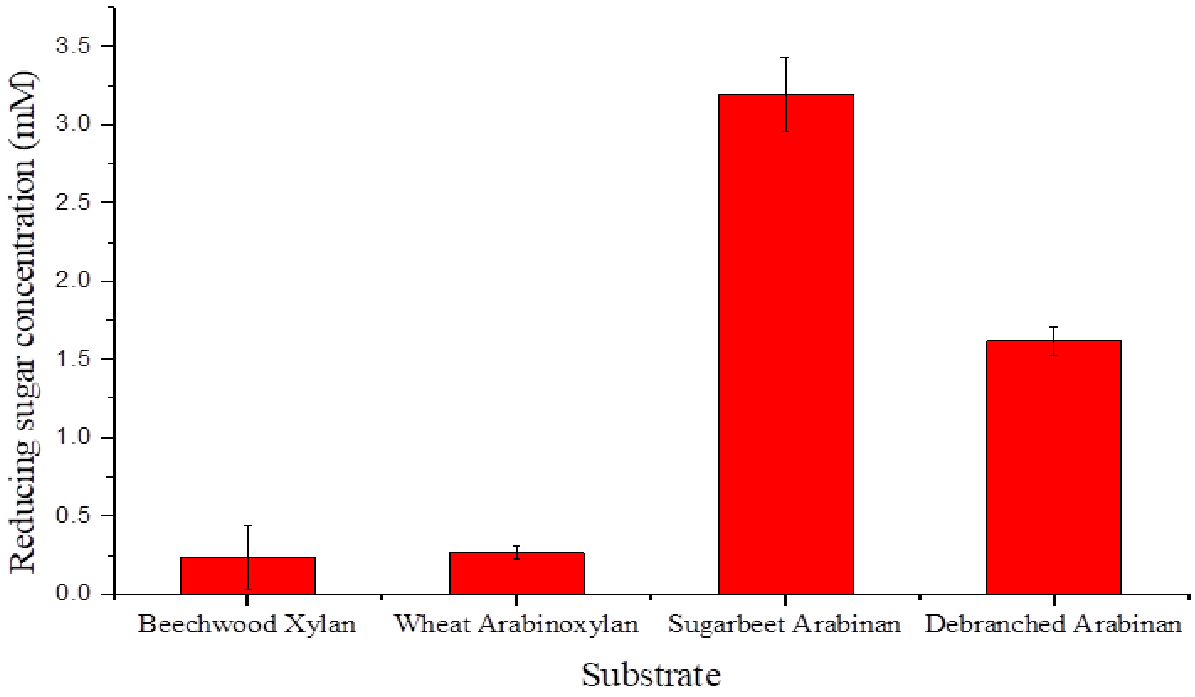

Fig. 4: The quality assessment of the hypothetical protein, (A) the per residue secondary structure of the predicted protein model, (B) ERRAT score of the model score.

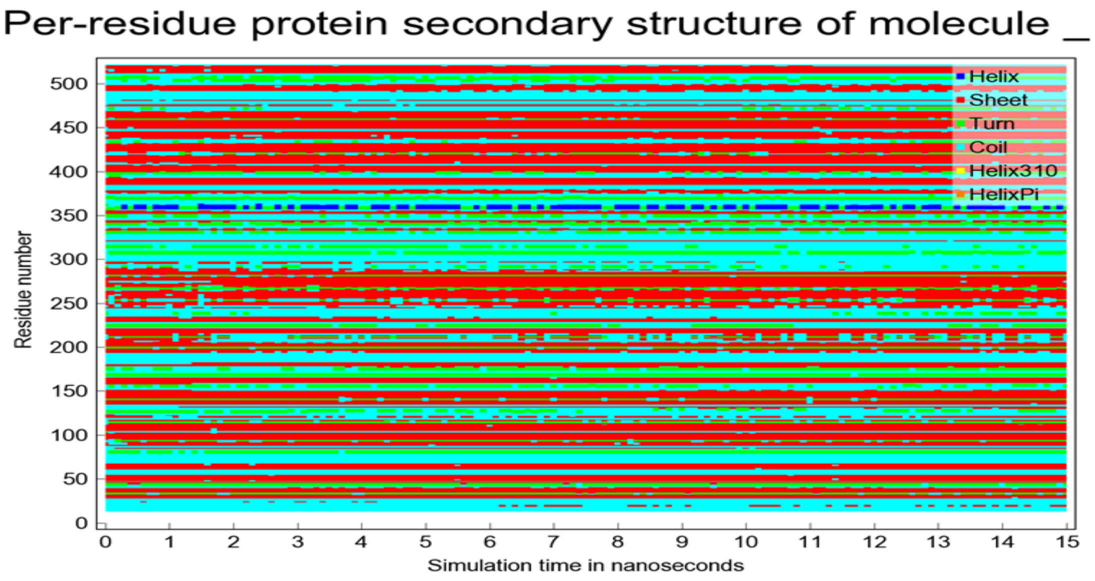

(A)

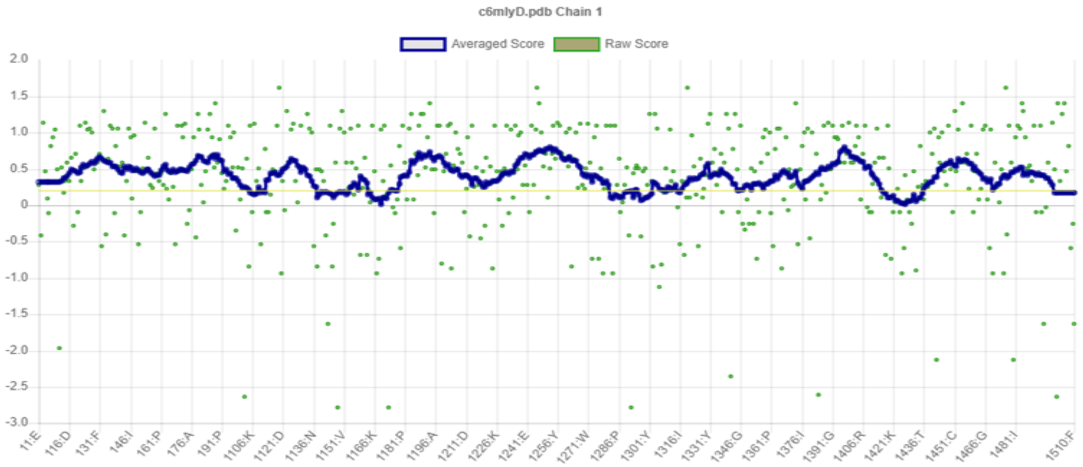

(B)

.

Fig. S5.Ramachandran map of modeled CAX43 protein.

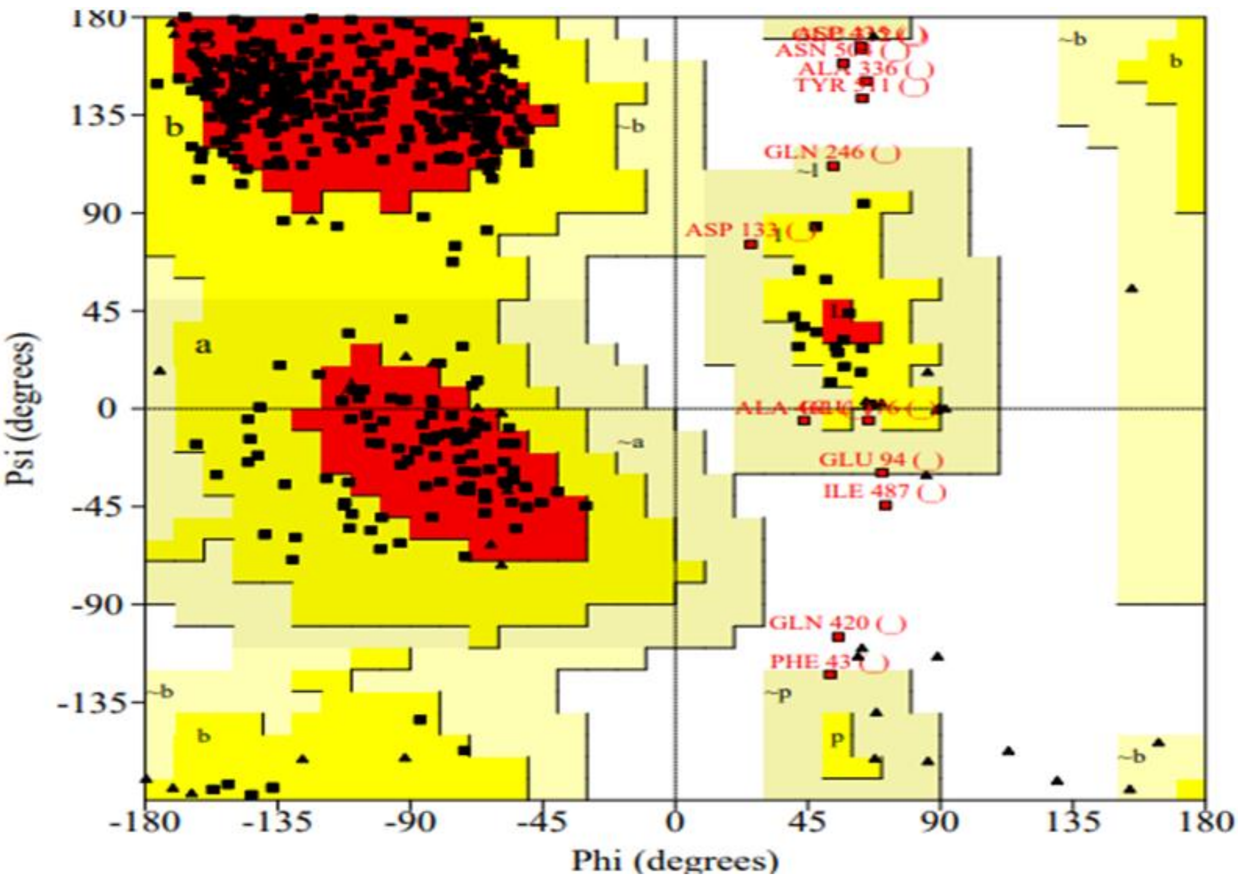

Fig. S6. The molecular dynamics simulation of the docked complexes; (A) root mean square deviation of the docked complex, (B) solvent accessible surface area of the complex, (C) radius of gyration of the complex, (D) hydrogen bond of the system.

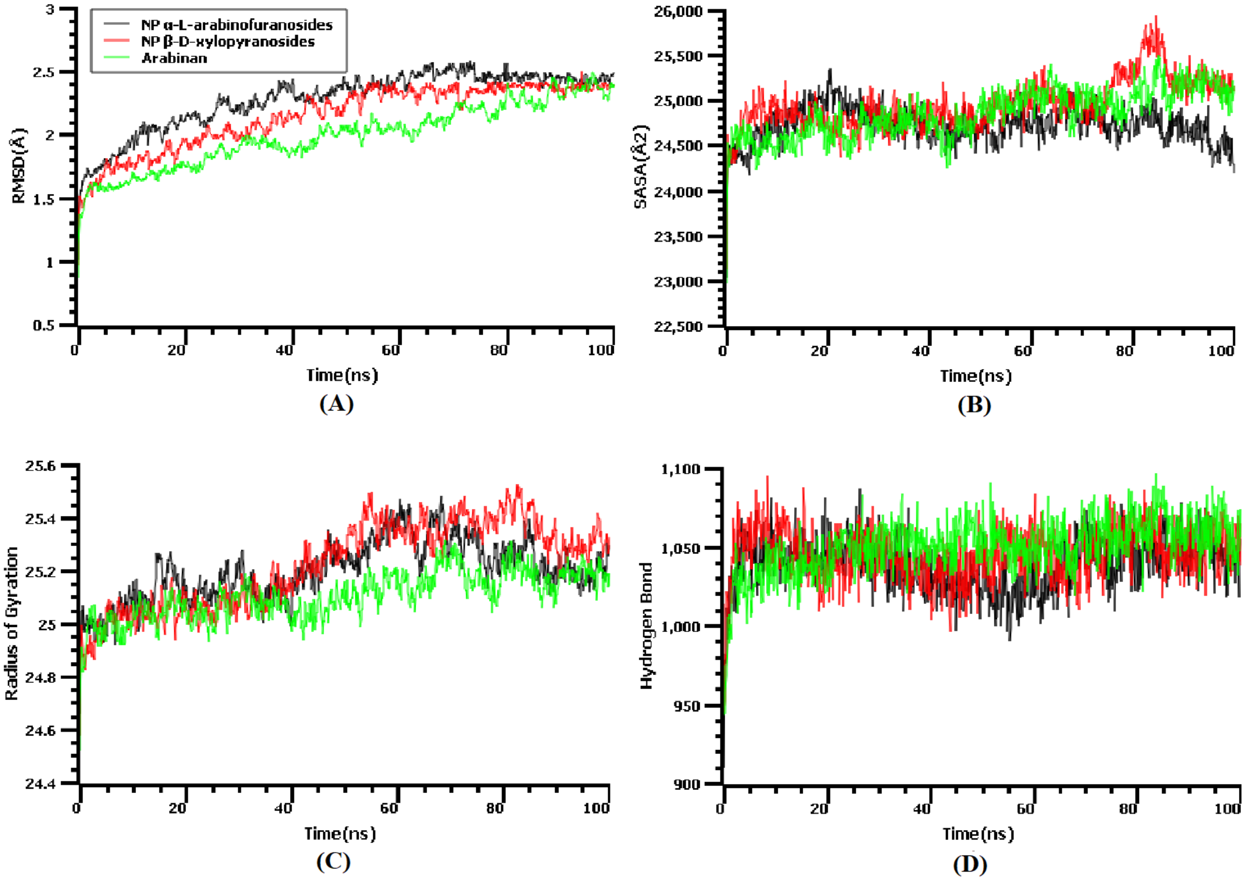

Fig. S7: The root mean square fluctuations of the docked complexes, where flexibility were observed for the across the residues in simulation trajectories.

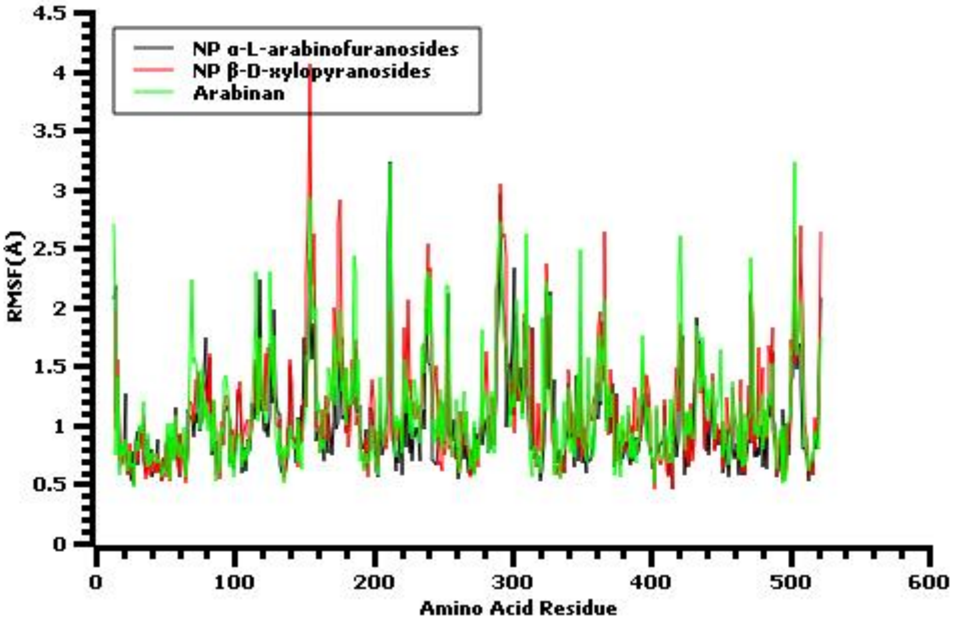

Supplement: Supplementary file 1 [file DataSheet1.pdf]
